# Modelling how incorporation of divalent cations affects calcite wettability–implications for biomineralisation and oil recovery

**DOI:** 10.1038/srep28854

**Published:** 2016-06-29

**Authors:** M. P. Andersson, K. Dideriksen, H. Sakuma, S. L. S. Stipp

**Affiliations:** 1 Nano-Science Center, Department of Chemistry, University of Copenhagen, Denmark; 2 National Institute for Materials Science, Japan

## Abstract

Using density functional theory and geochemical speciation modelling, we predicted how solid-fluid interfacial energy is changed, when divalent cations substitute into a calcite surface. The effect on wettability can be dramatic. Trace metal uptake can impact organic compound adsorption, with effects for example, on the ability of organisms to control crystal growth and our ability to predict the wettability of pore surfaces. Wettability influences how easily an organic phase can be removed from a surface, either organic compounds from contaminated soil or crude oil from a reservoir. In our simulations, transition metals substituted exothermically into calcite and more favourably into sites at the surface than in the bulk, meaning that surface properties are more strongly affected than results from bulk experiments imply. As a result of divalent cation substitution, calcite-fluid interfacial energy is significantly altered, enough to change macroscopic contact angle by tens of degrees. Substitution of Sr, Ba and Pb makes surfaces more hydrophobic. With substitution of Mg and the transition metals, calcite becomes more hydrophilic, weakening organic compound adsorption. For biomineralisation, this provides a switch for turning on and off the activity of organic crystal growth inhibitors, thereby controlling the shape of the associated mineral phase.

The interfacial energy, between mineral-water and mineral-organic compound, is perhaps the property that most strongly influences mineral growth on organic templates^1^. Organisms can control biomineralisation by taking advantage of differences in interfacial energy, to form intricately shaped crystals^2^,^3^. Atomic force microscopy experiments on calcite in solutions containing complex polysaccharides demonstrated that behaviour of the organic material, and thus crystal form, is controlled by the presence of trace divalent cations^4^. The ability to predict surface energy for minerals in equilibrium with various solutions of organic compounds and trace elements would open up new routes for bioinspired material design. The solid-fluid interfacial energy also determines the overall wettability of pore surfaces in soil, sediments and rocks and controls how oil, water and gas adsorb on mineral surfaces or flow through pore networks. The surface energy thus determines how difficult it is to remove organic contaminants from soil and how much oil can be produced from carbonate reservoirs. Some carbonate rocks are mixed wet, such as chalk^5^, while the more crystalline limestone reservoirs are significantly more oil wet^6^. In both cases, organic phases are more easily released if the wettability can be altered toward more hydrophilic pore surfaces. Pore network modelling suggests that a change in contact angle of about 10° toward more hydrophilic conditions would be enough to release ~10% more oil^7^ in response to water injection. In this paper we focus on how pore surface composition influences the wettability. Thus our study has isolated the effects of composition on a smooth surface from the important contribution of surface topography, i.e. roughness.

Imbibition experiments with chalk^8^ demonstrated that the presence of Mg in the water phase could significantly change the wettability of the rock, in particular when the solution also contained SO_4_. The adhesion between a hydrophobic tip and calcite at 70 °C was significantly lower in the presence of MgSO_4_ than in NaCl, consistent with a more hydrophilic surface. Density functional theory calculations for trace element uptake by calcite provide an explanation for the phenomenon^9^,^10^. Adsorption energy of single molecules to the mineral surface is strongly affected by Mg ion incorporation into the calcite surface. The adsorption energy difference is large enough that the macroscopic wettability is affected^9^. Mg incorporation into calcite is endothermic^9^,^11^, which means that dissolved Mg concentration must be about 10 times higher than Ca concentration to induce a change in composition, thus wettability. Density functional theory calculations for other divalent metals (Sr, Ni, Cd and Pb) predicted that while Sr incorporation is endothermic, as for Mg, the incorporation into calcite of Ni, Cd and Pb is exothermic^12^ and their sorption, which changes the surface behaviour of calcite, occurs at even lower concentrations than for Mg and Sr.

It is well known that trace metals are taken up on and in the crystal structure of calcite and the extent of uptake varies depending on the ion and the solution conditions. For example, Fe and Zn can be taken up by biogenic calcite and they significantly affect coccolith formation^13^. In a dominantly inorganic system, Olsson *et al*.^14^ measured trace element concentration in calcite that had formed as travertine after the Eyjafjallajökull eruption and observed a clear correlation of the solid:solution partition coefficients, P_Me_, with literature data. In general, the calcite P_Me_ for the alkaline earth metals, Mg^15^, Sr^15^ and Ba^16^ is <1, for Ni^17^ it is ~1, while P_Me_ for Cd^18^ and the transition metals Mn^19^, Fe^19^, Co^18^, Cu^20^ and Zn^20^ is >1. The general rule of thumb is that ions the same size or smaller than Ca, i.e. Mg, the transition metals and Cd, thus readily incorporate into calcite whereas for those that are larger, Sr, Ba and Pb, incorporation is limited. Little is known however, about how the change in surface composition as a result of adsorption or substitution affects the energy of the solid-fluid interface or how the surface preference for organic molecules over water changes, when trace metals are sorbed by calcite. Trace metals are certainly present in systems where biomineralisation takes place but their role is largely unknown. Anions can also change surface wettability but in this study, we focused on cations.

In chalk and limestone, recrystallized, rhombohedral calcite particles are present, which indicates that at least a portion of the pore surfaces consist of calcite, particularly {10.4} faces. If we can change the surface energy of these faces locally, we can change the overall contact angle of the material because we change the average surface energy. The cations Mn, Fe, Co, Ni, Cu and Zn are often present in environments where biomineralisation takes place. Mn, Fe and Zn are abundant enough to be considered as cost effective candidates for use in water induced, enhanced oil recovery (EOR) in carbonate reservoirs. Sr and Ba are interesting because they are present in sea water at low concentrations, thus of interest in biomineralisation, and Pb and Cd are examples of divalent ions that are quite toxic, and whose migration in calcite containing soil is important from an environmental aspect. Ni, Co, Cu and Zn are also interesting from the perspective of heavy metal contamination in soils or the leachate from mine tailings. Systems where biogenic calcite forms and fluids flow in the pores of soil and rock are very complex, where conditions can vary widely, including parameters such as temperature and composition: of the water, the minerals, the organic compounds and the gases present. We have taken some of these parameters into account in our simulations, in an attempt to make more informed suggestions about which ions would have the most influence in biomineralisation and about the best candidates for wettability modification in rock formation, depending on the system conditions.

The goal of this work was to use a combination of density functional theory (DFT) and geochemical speciation modelling to make a computational reconnaissance, to predict how much calcite wettability is altered by some of the divalent cations, under conditions that are realistic in natural systems. As the model surface to represent chalk and limestone reservoirs, we used the most stable calcite face {10.4}. In addition to being observed on pore surfaces in aquifers and oil reservoirs, this face is also observed on coccoliths^4^,^21^,^22^, making our results relevant for biomineralisation as well. We have used plane-wave DFT to determine adsorption properties of model compounds on pure and cation substituted calcite and derived corresponding wettability changes in terms of contact angle from the calculations. We have also used DFT calculations with the COSMO-RS implicit solvent to calculate the composition of the oil-water interface and pK_a_ of carboxylic acids adsorbed on calcite. This is potentially important because in calcium carbonate systems, pH is generally between 8.3 and 10.3, higher than the pK_a_ of carboxylic acids so the acids would be mostly deprotonated. The impact of metals on calcite wettability relies on their general association with calcite surfaces. Adsorption would not be possible if they instead were to form separate stable phases. To predict solubility for the divalent metals in systems where calcite is present at ambient conditions and in the deeper subsurface, we used geochemical modelling, with the speciation program, PHREEQC^23^. This software allows calculation of the aqueous speciation and mineral solubility at equilibrium based on thermodynamic data from databases that have been tested for consistency. By combining the various computational techniques, we were able to extrapolate our vacuum DFT calculations to more realistic conditions, to account for substitution and precipitation effects as a function of pH and temperature.

## Computational Details

### Plane wave density functional theory (DFT) calculations

All DFT calculations for adsorption energies on calcite were made using the Quantum Espresso package^24^ in a plane wave implementation. We used the Perdew Burke Ernzerhof (PBE) functional^25^ with ultrasoft pseudopotentials taken from the Quantum Espresso pseudopotential library: Ca.pbe-nsp-van.UPF, C.pbe-rrkjus.UPF, O.pbe-rrkjus.UPF, H.pbe-rrkjus.UPF, Mg.pw91-np-van.UPF, Sr.pbe-nsp-van.UPF, Cu.pbe-d-rrkjus.UPF, Ni.pbe-nd-rrkjus.UPF, Fe.pbe-nd-rrkjus.UPF, Co.pbe-nd-rrkjus.UPF, Mn.pbe-sp-van.UPF and Zn.pbe-van.UPF. We used a kinetic energy cutoff at 25 Rydberg and a density cutoff at 250 Rydberg. These parameters gave converged adsorption energies for water on the original, unsubstituted calcite slab.

**Figure 1:**
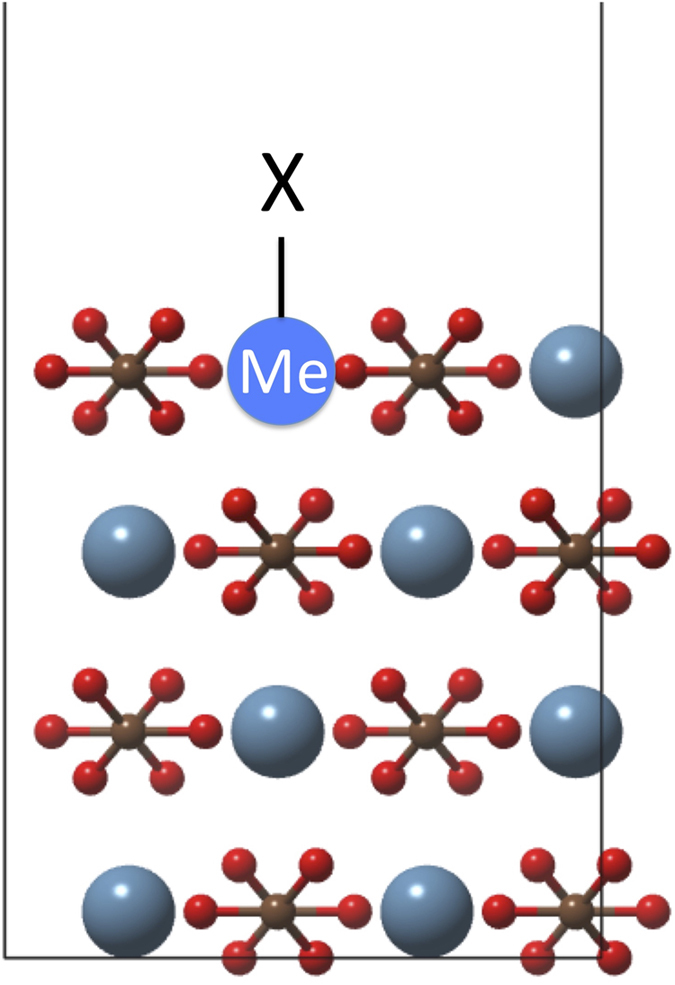
Side view of the calcite slab used in the DFT calculations. Me denotes a substituted ion and X, an adsorbed molecule.

We used spin polarised calculations for all systems with copper (doublet), nickel (triplet), cobalt (quartet), iron (quintet) and manganese (sextet). The calcite {10.4} slabs were created using the lattice parameters of bulk calcite derived from X-ray diffraction and reoptimised using a kinetic energy cutoff of 35 Ry and a density cutoff of 350 Ry. These gave lattice parameters that converged to within 0.01 Å. The slabs were four molecular layers thick, with a simulation cell equal of 1 × 2 primitive calcite unit cells (80 atoms), i.e. four metal atoms and four carbonate units per molecular layer (Fig. 1). The atoms in the lowest molecular layer in the calcite slab were fixed at bulk positions during the optimisation to represent an essentially infinite solid. At least 15 Å of vacuum was present between the slabs to minimise interactions between them.

In the calculations for ion substitution energy and adsorption energy for water, all slabs had a monolayer of water adsorbed, which amounts to one water molecule per surface cation. The differential adsorption energy, which can be viewed as a discretized version of the chemical potential, is equal for the first four water molecules on pure calcite surfaces and significantly stronger than for subsequent layers, thereby clearly defining a monolayer of water as one water molecule per surface cation^9^. The solvent beyond the first monolayer of water was disregarded, because for a subset of the ions studied here, the free energy of solvation of a calcite surface with a monolayer of water was independent of ion substitution^12^. Geometry optimisation used only the gamma point, because single point energies with a 2 × 2 × 1 Monkhorst Pack k-point mesh^26^ confirmed that gamma point adsorption energies for water converged within 0.01 eV. We also included empirical dispersion using the DFT-D2 approach^27^ because of increased accuracy of adsorption energies on inorganic solids predicted with DFT^28^,^29^,^30^. This is particularly important for interactions of benzene or other hydrophobic species with calcite, which are dominated by dispersion forces.

All calculations for hydrated ions, free ions and water were made in a cubic box with dimensions 12 Å on a side and we used the Martyna-Tuckerman correction^31^ to determine energies for isolated charged systems in a periodic plane wave calculation.

### COSMO-RS calculations

COSMO-RS is an implicit solvent model, which uses DFT to generate a screening charge surface, divided into segments with a chosen area and charge. All solvent interactions are handled using segment-segment interactions and include the effect of hydrogen bonding, dispersion and electrostatic effects. In addition to providing thermodynamic properties such as phase diagrams, solvation energies and solubilities, COSMO-RS can be used to determine pK_a_ values very effectively, by calculating the free energy difference between the protonated and the deprotonated form of a molecule. The composition of the oil-water interface and the pK_a_ for water and acetic acid adsorbed on substituted calcite slabs were calculated using COSMO-RS^32^ and the COSMOtherm^33^,^34^ program. We used Turbomole v6.5^35^,^36^, the BP^37^,^38^ functional and the TZVP basis set^39^ and an infinite dielectric constant for the COSMO implicit solvent. An infinite dielectric constant is a requirement for the COSMO-RS calculations because a common reference state, regardless of the solvent, is needed to ensure that all solvent interactions are taken care of by the screening charge surfaces. The BP_TZVP_C30_1301 parameterization was used in all COSMO-RS calculations. For the surface pK_a_ calculations, we used 80 atom clusters to represent the calcite surface and relaxed only the 10 atoms of the surface that are not located at an edge (Fig. SI-1). This procedure gave a converged pK_a_ for water on calcite^40^. pK_a_ predictions from COSMO-RS have a RMS error of 0.5 pH units in solution, where the method is parameterized. It is difficult to assess the corresponding accuracy at surfaces but the error is probably slightly higher. The flatsurf module of COSMOtherm was used to estimate the composition of the oil-water interface, similar to our previous study^41^, with an oil-water interfacial tension of 17.1 mN/m.

### Adsorption energies

The binding energy, Eb, of water molecules to the divalent cations in vacuum was determined using:


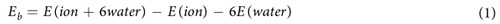


This definition means that the more negative the binding energy, the more strongly the water molecules attach to the ion.

For water and a set of organic molecules, we have also calculated the adsorption energies on calcite, E_ads_:


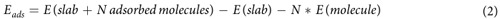


All organic molecules adsorbed on the substituted ion, where there was one, and N = 1. We calculated the adsorption energy of a single water molecule on the substituted slabs from the monolayer (ML) calculations with 4 water molecules, to keep the results for water consistent with those of the previous study^9^ and because the first monolayer of water is a good model for full hydration on calcite:


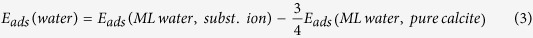


This procedure can be used because the differential adsorption energies are the same for the first four water molecules on the original calcite and the three water molecules that remain bound to the surface Ca ions after Mg has substituted on the calcite slab^9^.

To compare the adsorption energies for molecules adsorbed on substituted slabs to the same molecule adsorbed on pure calcite, we have determined differences in adsorption energy, ΔEads, for the same molecule:


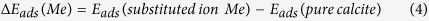


To make changes in wettability easier to quantify, we have also calculated the change of the difference in adsorption energy for organic molecule X, relative to water:


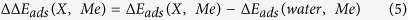


This quantity, ΔΔE_ads_(X, Me), tells us how the surface preference for organic compound X compares with its preference for water and how this changes when a surface Ca^2+^ is substituted with another divalent cation, Me^2+^. If ΔΔE_ads_(X, Me) is negative, the preference of X to adsorb on the surface compared with water is higher after Me has exchanged than on the initial calcite.

### Ion substitution

The exchange reaction we used to estimate the energy for ion substitution is^9^,^12^:


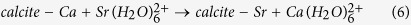


where calcite-Ca is an unsubstituted calcite slab covered by a monolayer of water and calcite-Sr is a calcite slab with a monolayer of water where one Ca^2+^ ion is substituted by a Sr^2+^ ion. The reaction is analogous for substitution of all the divalent cations. We use the reaction energy from Eq. 6 as an approximation of the free energy of the substitution reaction. Most of the effects of entropy are expected to cancel because the two sides are very similar. The entropy of various metal carbonates is also similar (for example Table 3A in ref. 42), which further supports our assumption. Configurational entropy is disregarded because it is the same for all substituting ions. It would always favour some small degree of incorporation, regardless of the substitution energy.

It is worth mentioning that the concentration of foreign ions in our calculations is rather high because of the need to use a limited number of atoms and the periodicity of the simulation cell. Therefore we limit our interpretations to surfaces with a maximum of 25% substitution because only 1 out of 4 cations in the xy plane is substituted. Lattice strain effects are smaller for lower concentrations, which would only make the substitutions more favourable than our predictions suggest.

### Estimating change in contact angle

The predicted change in contact angle can be deduced from Young’s equation using the changes in adsorption energy predicted by DFT and the area per substituted ion^9^:


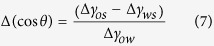


The subscripts denote o = oil, w = water and s = surface. We have used a value of 17.1 mN/m for the oil-water interfacial tension, γ_ow_^9^, which is a reasonable value for the interface between crude oil and brine^43^. The numerator is evaluated from:


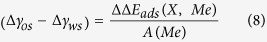


where A(Me) represents the area per substituted ion, which is estimated from the dimension of the surface unit cell and the number of substitutions per unit cell calculated from ion concentrations and ion substitution energies from Eq. 6.

### PHREEQC calculations

In addition to being associated with the calcite surfaces, the divalent cations can also form discrete phases. The solubility of these phases would limit their aqueous concentration and hence their adsorption density on calcite. To estimate the metal concentration in equilibrium with sulphide and carbonate minerals, calculations were made with the geochemical speciation code PHREEQC^23^, with the llnl.dat database from Lawrence Livermore National Laboratory^44^, which was adapted from the SUPCRT database^45^. llnl.dat does not include an equilibrium constant for the dissolution of CuCO_3_. Consequently, we used a value from the database wateq4f.dat. The data in the databases have been evaluated and consistency among them has been assured but for calculations with data for Cu^2+^, we assume higher uncertainty.

All calculations were initially made for all cations and were constructed with pure water, temperature of 25 °C and pressure of 1 bar, where PHREEQC determines aqueous speciation using a modified version of the extended Debye-Hückel equation to account for the nonideal behaviour of the aqueous species^46^.


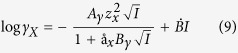


where the values for Aγ, Bγ, z^2^_x_, å_x_ and 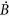 are included in the llnl database. γ_x_ refers to the activity coefficient which relates ion activities (a_x_) with their concentrations (C_x_):


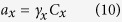


and I refers to the ionic strength of the solution, which is calculated from the concentration of the species and their charge (z_x_):


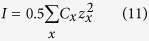


The conditions in these calculations clearly differ from those of a reservoir, where temperature, pressure and salinity are much higher, or from a contaminated aquifer where temperature is lower and ionic strength can vary over a few meters or in a biomineralising system where ionic strength can be quite high. Thus, the calculations are estimates, rather than precise predictions of aqueous metal concentrations.

We performed calculations, which fall into two groups:

- Carbonate minerals as the solids; CO_2_, present at partial pressure ranging from 1 to 10^−3.5^ bar or absent, such as in a closed system.
- Carbonate and sulphide minerals present; CO_2_ and H_2_S at partial pressure ranging from 1 to 10^−3.5^ bar, either both present or both absent, as for a closed system.

In all calculations, there was an excess of calcite (CaCO_3_). We also included 0.1 M of the following phases in calculations for the conditions of Set I: calcite (CaCO_3_), siderite (FeCO_3_), magnesite (MgCO_3_), sphaerocobaltite (CoCO_3_), cupric carbonate (CuCO_3_), rhodochrosite (MnCO_3_), gaspeite (NiCO_3_) and smithsonite (ZnCO_3_). Calculations for the conditions of Set II included the sulphide phases in addition: pyrrhotite (FeS), cobalt sulphide (CoS), covellite (CuS), alabandite (MnS), millerite (NiS) and sphalerite (ZnS).

It should be noted that iron sulphide occurs in sediments most commonly as pyrite, not pyrrhotite, which we have used in our model. Pyrite dissolution/precipitation requires electron transfer:


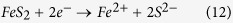


meaning that including pyrite in the model would require knowledge of reservoir redox conditions. Measurement of redox potential in deep groundwater, such as an oil reservoir, is complicated (e.g., ref. 47). Redox sensitive species from which redox potential could be calculated, such as C and S, feature a volatile phase redox state that is prone to degas from solution prior to sampling, and redox couples can be in disequilibrium or in metastable equilibrium (e.g. refs 42,48). Consequently, we have not included pyrite. Such calculations are far beyond the scope of this paper. Nevertheless, our calculations provide useful information about solubility issues with and without the presence of S^2−^. In particular, they can help identify the trends across the range of cations investigated here.

Significant amounts of trace metals can be incorporated in silicate minerals. However, these phases are generally low in abundance in systems where carbonate minerals are at equilibrium so their contribution to controlling the metal concentration is negligible.

In addition to the above calculations at 25 °C and low ionic strength, PHREEQC simulations of a carbonate system with Ca^2+^ and Mn^2+^ were conducted at reservoir conditions (i.e., high salinity, temperature and pressure) to model the injection of the metal into a limestone reservoir. Because of the high ionic strength of these solutions, the nonideal behaviour of aqueous ions was accounted for using the semiempirical Pitzer approach, which relies on description of ion interactions by temperature dependent virial coefficients (e.g. refs 49,50. In this modelling, we used the native Pitzer database of PHREEQC, compiled by Plummer *et al*.^51^.

## Results and Discussion

### Hydration energy

**Figure 2:**
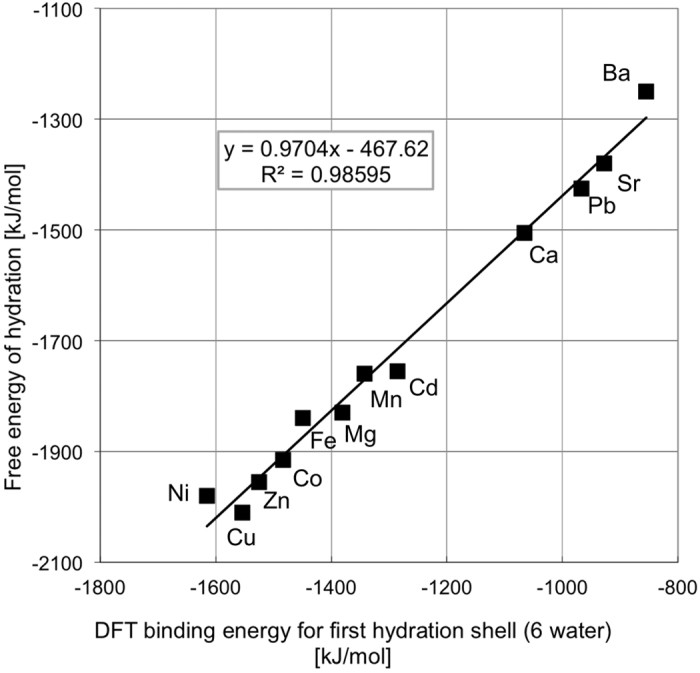
Free energy of hydration vs the DFT binding energy for the first hydration shell (6 water molecules) for the divalent cations.

To test if our model is robust, we compared our simulation results for binding energy of six water molecules to cations (Equation 1) with experimental values for free energy of solvation^52^ (Fig. 2). As in previous studies^9^,^12^, the straight line with a slope close to 1 confirms the validity of using the DFT binding energy as an accurate descriptor for the free energy of hydration, also for the first row transition metal ions. It implies that in Eq. 1, differences in DFT energy reflect differences in free energy. While the uncertainties in individual differences are on the order 10–20 kJ/mol, energy differences for ion substitution energy calculations are probably smaller because errors cancel. For example, if the bonding of a particular cation to six oxygen atoms in water molecules is too strong, it is probably also too strongly bound to six oxygen atoms in a hydrated calcite surface. The majority of the constant offset of 467 kJ/mol results from neglecting solvent effects beyond the first hydration shell. Because we are only interested in energy *differences* between ions, the offset is irrelevant in our case.

### Divalent metal ion substitution energy

The ion substitution energies, presented in Table 1, show that all transition metals, as well as Cd and Pb, substitute exothermically into calcite, which is consistent with the lower solubility of the carbonate minerals of these cations, compared with calcium, magnesium and strontium carbonates. Our predicted enthalpies for the cation substitution reaction are also consistent with experimental partition coefficients, P_Me_, for the incorporation of these ions into calcite^14^. The ions, for which we predict endothermic substitution reactions have P_Me_ <1, and the ions for which we predict exothermic reactions, P_Me_ >1. The only significant deviation from this behaviour is observed for Ba, which has P_Me_ <1 but for which our calculated substitution energy is negative. Too negative substitution energies for the heavy ions result from the treatment of dispersion forces. The parameters in the Grimme DFT-D2 dispersion treatment do not consider oxidation state and cations in ionic materials have dispersion parameters that are about an order of magnitude lower^30^,^53^. The choice of pseudopotential plays only a minor role but could impact the substitution energies by up to 9 kJ/mol, which is the difference in the substitution energy for Mg in this work compared with our previous work^9^, which used a different set of pseudopotentials.

Our results offer interesting insight, in that nearly all ions prefer incorporation in the surface layer rather than in bulk sites. This makes sense because ionic radius and strong hydration are less important if the ion only has to fit an ordering of 2 dimensions rather than 3, as in the bulk. The change in surface behaviour is therefore significantly higher than what would be deduced from macroscopic, batch experiments based on bulk composition. There are only two deviations from this behaviour: Ba, where the difference could equally well be an artefact in the simulations from the dispersion treatment, and Cd, which is known to form a complete solid solution with calcite^54^. There is also evidence that Cd is more stable in the bulk than at the surface^55^.

### Dehydration

The temperature above which ion substitution into calcite can proceed is an important parameter to take into account, particularly for organisms that live at sea water temperatures and low temperature reservoirs, where substitution of strongly hydrated ions might not take place in a reasonable amount of time, during life processes or during a water flood in an oil reservoir. Experiments demonstrate that Mg uptake by calcite is strongly temperature dependent. Magnesium is only able to dehydrate when T > ~70 °C^56^. Calcite dissolution and precipitation, which require dehydration, occur readily at sea water temperature. Molecular dynamics (MD) simulations demonstrate that the sea water ions, Sr and Ba, exchange water in the first hydration shell as easily as Ca^57^. Ni is the only ion in our study that dehydrates more slowly than Mg^58^, whereas Co, Mn, Fe, Cu and Zn dehydrate more rapidly than Mg^58^,^59^. This means that in low temperature reservoirs, Mg and Ni ion incorporation are most likely kinetically hindered.

### Adsorption energy

The adsorption of organic molecules on calcite modifies the wettability of the surface, especially when the molecule adheres through a polar group and a hydrophobic end sticks out into solution. This would then act as an anchor point for other hydrocarbons, rendering the mineral surface more hydrophobic^60^,^61^. The relative preference for water over organic molecules describes how much the wettability of the calcite surface changes when ions are substituted. Table 2 presents adsorption energy for water, acetic acid, pyridine, phenol and benzene. Organic molecules bind to calcite, with and without transition metals substituted into the surface layer, in the order:


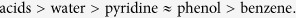


Acidic polysaccharides are often part of the organic material associated with marine organisms, such as coccolithophores. Specific adsorption of carboxylic acids to calcite has been suggested to play a major role in crystal growth in general, including biomineralisation rates^62^ and the presence of cations affects their bonding character^4^. Also at oil-water interfaces, carboxylic acids are significantly more surface active than basic functional groups and alcohols^41^.

At conditions relevant for sea water organisms and carbonate reservoirs, pH is >8 so carboxylic acids are deprotonated but in this work, we only consider adsorption energies of protonated acids to maintain neutrality in our unit cell. We have previously shown that the pK_a_ of water changes when it binds to calcite^40^ and the inclusion of a foreign ion would also be likely to alter the pK_a_ of adsorbed water and carboxylic acids. In the Supplementary Information section, we present DFT calculations for pK_a_ of water and acetic acid on pure calcite and calcite where an ion has substituted in the surface layer. The pK_a_ for water is in all cases >9.4, which means that water is more stable than hydroxyl ions on all surfaces. The pK_a_ for acetic acid differs at most by 1.6 pH units for the transition metals, which corresponds to less than 10 kJ/mol at 298 K. This shows that our predictions about energy differences for substituted and unsubstituted calcite, using the neutral carboxylic acids, can be assumed to be valid for adsorption of the acid anions as well, in the low substitution limit. Presented in Table 3 is the difference in adsorption energy between organic molecules, denoted X, and water during cation substitution, i.e. ΔΔE(X,I) (Equation 5), which reflects the change in wettability when ions are sorbed.

### Composition of the oil-water interface

The composition of the oil-water interface predicted by COSMO-RS is presented in Table 4. The corresponding values for the free energy of transfer are in Table SI-3. Acids are enriched to a great extent at the oil-water interface, much more than alcohols, bases and aromatic hydrocarbons. Only when the bulk concentration of a nitrogen base is ten times higher than that of the acid is surface concentration comparable. In considering wettability changes, we assume that the composition of this interface between oil and polar water also reflects the composition at an oil interface with polar calcite.

### Wettability changes on an ion by ion basis

**Figure 3:**
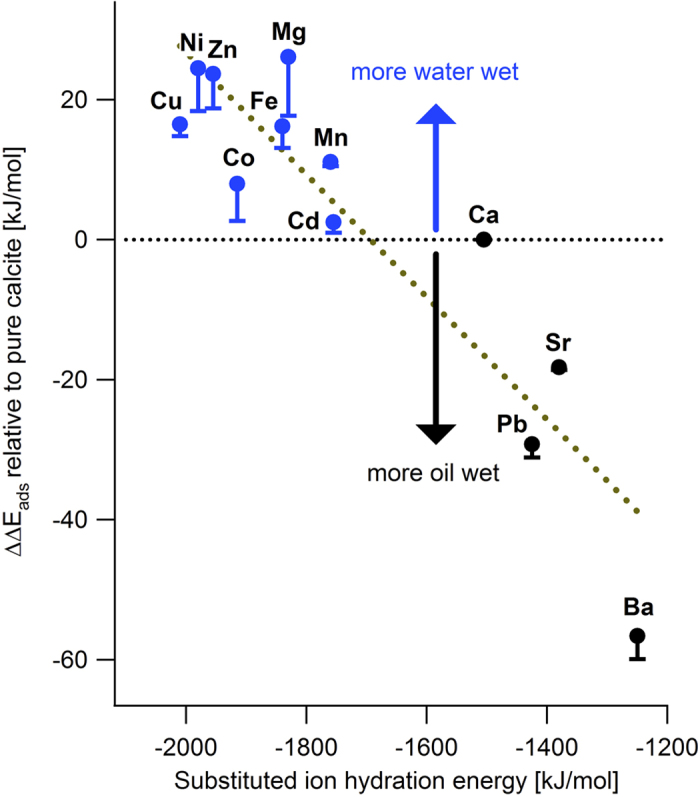
Predicted wettability change (Eq. 5) as a function of ion hydration energy resulting from substitution in a calcite {10.4} surface. The points show values obtained for a model oil that contains 0.1 mol% phenol, acetic acid and pyridine (Table 4). The bars show the difference in results using the most oil wet case of a high acid oil with 1% phenol and acetic acid or a high base oil with 1% pyridine. The dashed line is the best linear fit to the data.

We now know how cation substitution and the composition of the oil-water interface affect the adsorption energy for organic molecules relative to water. This means that we are now able to discuss how calcite wettability changes as a result of cation substitution and adsorption of a model organic compound. Figure 3 shows a plot of composition averaged ΔΔE(X, Me), as a function of ion hydration energy. To derive composition averaged ΔΔE(X, Me), we weighted the values of ΔΔE(X, Me) in Table 2b by the composition predicted at the oil-water interface (Table 4). Figure 3 clearly shows that all of the transition metals considered in this study are predicted to make the calcite surface more water wet in the presence of organic molecules, even those that have high acid numbers or high base numbers. The predicted change in wettability was directly correlated with the substituted ion hydration energy (Fig. 3). Ions that are hydrated more strongly than Ca make calcite more hydrophilic, whereas ions that are more weakly hydrated than Ca make calcite more hydrophobic. This is a quite general and quite intuitive result. It will be interesting to see if it can be extended to other mineral surfaces than calcite.

An important conclusion from these results is that the first row transition metals and Mg alter the wettability on calcite surfaces to more water wet. Uptake of Sr and Ba renders calcite less water wet. This behaviour offers interesting possibilities for marine organisms to modify the surface properties of biogenic calcite simply by controlling the concentration of the fluid that they allow to be in contact with the crystal they are producing. It also offers interesting potential for enhancing oil recovery (EOR) in carbonate reservoirs. The substitution energy for the transition metals is exothermic so only small concentrations would be required for uptake to reach levels that are high enough to produce substantial effects. In the case of EOR, reservoir temperature is an important parameter to consider because Ni, which is predicted to strongly alter wettability, might require a temperature higher than Mg to dehydrate^58^.

The fact that trace metals strongly affect mineral-fluid interfacial energies is also very important for a detailed understanding of biomineralisation in natural waters, where trace metals are certainly present. The exothermic substitution into calcite and the significant change in wettability could be an integral part of an organism’s ability to form minerals with the composition, form and structure required by the organism. Significant changes in mineral-fluid interfacial energies could be part of the explanation for why changes in Fe and Zn concentration so strongly affect biogenic calcification in coccolithophorids^13^. This is important from the perspective of global environment because the health of these species of algae has implications for CO_2_ partitioning between the atmosphere, biogenic calcite and the ocean and thus, O_2_ production.

### The effect of CO_2_ and H_2_S on cation concentration

The divalent metals may form a variety of phases, whose solubility can limit their aqueous concentrations and thereby their surface density on calcite. Calcite is a major component of limestone reservoir formations and they can also contain smaller quantities of sulphide minerals. Thus, both carbonates and sulphide solids are able to limit the solubility of the divalent metals. In addition, CO_2_ and H_2_S gases are present in some oil reservoirs, giving rise to solution-gas equilibria. The presence of CO_2_ and H_2_S would have a strong influence on pH and therefore, the concentration of dissolved carbonate and sulphide species, resulting in markedly different solubility for the transition metals. We excluded Cd from our calculations, because the predicted wettability change is negligible and Cd partitions to calcite more readily than to sulphide minerals. All of the divalent cations can form as carbonate compounds to some extent and the solubility of these minerals in a reservoir controls the maximum concentration that the pore fluids can tolerate. Precipitation of these carbonate minerals would be a disadvantage for EOR because it would diminish the positive effects of using it as an additive and it could block pore throats, thus reducing permeability.

**Figure 4:**
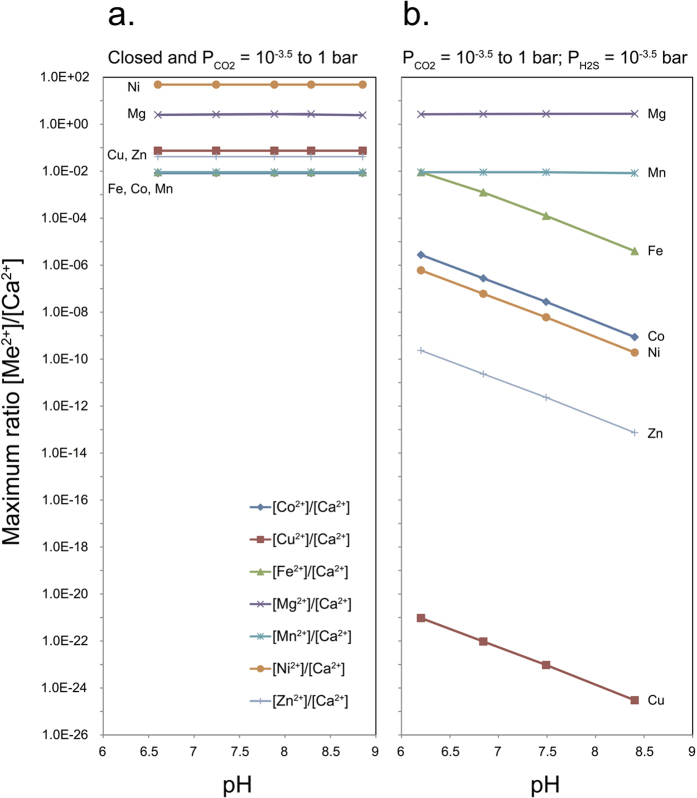
Estimates for the highest possible [Me^2+^]/[Ca^2+^] in a solution at equilibrium with (**a**) carbonate phases and CO_2_ (10^−3.5^ to 1 bar) and (**b**) carbonate and sulphide phases and gas phase CO_2_ (10^−3.5^ to 1 bar) and H_2_S (10^−3.5^ bar). pH is defined by the partial pressure of CO_2_.

We used PHREEQC to investigate the effect of divalent carbonate mineral solubility and the presence of CO_2_ and H_2_S on the maximum concentration of divalent metals that could be introduced into a reservoir to increase oil production. When only CO_2_ is present, we assume that all single metal carbonate minerals are also present. The equilibrium concentrations are shown in Fig. 4a. In this case there is no pH dependence in the concentration ratios Me^2+^/Ca^2+^ and all ions would be present in significant quantities to substitute into calcite and affect surface energy, thus the contact angle (Table 5). The transition metals incorporate into calcite at lower concentrations than where they begin to form separate carbonate phases, which is consistent with experimental data^14^,^63^. A closed system without CO_2_ gives the same [Me^2+^]/[Ca^2+^] ratios. All transition metals in our study increase water wettability so they are all possible candidates for water composition induced EOR. These results are consistent with the observation that CO_2_ concentration does not significantly influence coccolithophore calcification in the presence of Fe and Zn^13^.

In an oil reservoir where H_2_S is also present, in addition to CO_2_ (H_2_S at 10^−3.5^ atm and CO_2_ ranging from 10^−3.5^ to 1 atm), we considered the aqueous sulphide species and explored the control by metal sulphide minerals on divalent cation concentration. The results are presented in Fig. 4b. The solubility of Co, Ni, Zn and Cu sulphide phases are so low, that pore fluids at equilibrium with H_2_S would precipitate sulphide minerals, decreasing the concentration of free cations available for uptake by calcite to the extent that surface concentration on calcite would be negligible. The difference in free S^2−^ concentrations as a function of pH is what induces the pH dependence for ions with an insoluble sulphide solid phase. However, the sulphide phase of Mn is soluble and the sulphide phase of Fe is comparatively soluble so Mn and Fe can substitute into calcite. Fe and Mn exchange to a much greater extent than Mg, because of their exothermic substitution, which potentially leads to a larger change in wettability, Table 4. For higher pH solutions, divalent Fe concentration decreases and at pH > 7 (Fig. 4b), Mn becomes a better wettability modifier. Mn does not form a stable sulphide phase so it is available for uptake by calcite, in the studied pH range, which makes it a very good candidate for water EOR in carbonate reservoirs. We therefore extended our PHREEQC calculations to higher temperatures for Mn and Ca in order to better simulate solubility constraints at reservoir conditions.

Formation water in oil reservoirs is typically highly saline and pressure and temperature are considerably higher than standard conditions. The pore water in the Valhall chalk reservoir in the North Sea, for example, contains about 1 M NaCl^64^. Formation pressure is about 450 bars and temperature, ~90 °C^64^. To estimate an upper limit for Mn/Ca molar ratios in such solutions, geochemical modelling was performed with PHREEQC, using its Pitzer database. Mn^2+^ is not prone to formation of sulphides so we considered only the carbonate equilibria.

**Figure 5:**
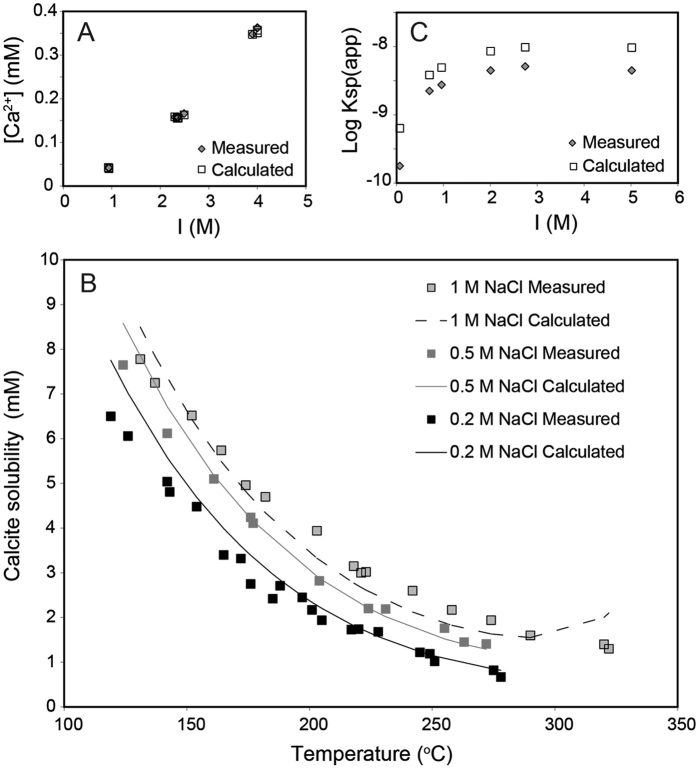
Comparison of PHREEQC modelling with experimental data for solubility of (**A**) calcite at 25 °C and high ionic strength^65^; (**B**) calcite at high ionic strength and elevated temperature^66^; (**C**) rhodochrosite at 25 °C and high ionic strength^69^.

First, we investigated the accuracy of the database by predicting calcite properties at reservoir conditions. The solubility was tested by comparing the Ca concentrations determined in equilibrium experiments^65^ at 25 °C and variable ionic strength with those calculated with PHREEQC (input file in SI). Figure 5A shows that there is excellent agreement between the experimental and predicted concentrations at 25 °C.

To probe the performance of the Pitzer database at higher temperature, calculated values (input file in SI) were compared with experimental data^66^ for calcite solubility at variable NaCl concentration and temperature. In the experiments, the resulting pressure had contributions stemming from an initial 12 bar of CO_2_ as well as that resulting from the heating. Except for a discrepancy at temperatures above 300 °C, the calculated and experimental solubilities compare favourably (Fig. 5B). Calculations at temperatures lower than 300 °C and higher pressure do not show an increase in solubility, suggesting that the discrepancy is not related to pressure increase alone. In conclusion, calculations with PHREEQC with the Pitzer database provide reliable results for calcite up to 300 °C.

The solubility of rhodochrocite, MnCO_3_, however, is much less well described. For example, reports of the solubility product of rhodochrocite at 25 °C and low ionic strength vary by about three orders of magnitude (e.g. ref. 67). In addition, the temperature dependence of the solubility constant is relatively poorly defined. Some experiments suggest increased solubility at 90 °C^67^, whereas the SUPCRT database^45^ predicts decreased solubility at 90 °C. Thus, considerable uncertainty is associated with the solubility product for rhodochrocite at the temperature releant for oil reservoirs, even without including effects from high ionic strength and pressure. The Pitzer database does not include solubility data for rhodochrocite so we used the solubility product (10^−10.08^), enthalpy of reaction (−1.764 kcal/mol) and molar volume (31.075 cm^3^/mol) from the 1998 version of the SUPCRT database^68^. Importing data from one database into another is somewhat risky because they may not be internally consistent. It would increase uncertainty in the calculations.

We modelled the apparent solubility product and compared with data from experiments^69^ (Fig. 5C). The agreement is good, in spite of the Pitzer database in PHREEQC not including Mn-carbonate complexes, which would also contribute uncertainty in the rhodochrocite solubility^67^. The average difference between predicted and experimental results is about a factor of 2 at an ionic strength of 0.96 for the pH interval 6.8 to 7.7. Based on this modelling, we expect the accuracy for the Pitzer parameters to be well within one order of magnitude at 25 °C, if the calculations are carried out at relatively acidic conditions to minimise the influence of Mn-carbonate complexation.

Unfortunately, we could find no experimental studies in the literature on rhodochrocite solubility at high temperature, pressure and ionic strength so the uncertainties for reservoir conditions are unknown. We expect them to be smaller than the uncertainty of the rhodochrocite solubility product itself. The solubility product from the 1998 version of SUPCRT^68^ is roughly in the middle of data from experiments. Thus, at 90 °C, we would expect error bars of about two orders of magnitude from the combined uncertainty at low ionic strength. The use of Pitzer parameters in the calculation could contribute an order of magnitude additional uncertainty.

Using reservoir conditions (1 M NaCl, 90 °C, 447 bar total pressure), CO_2_ partial pressure of 0.1 bar and equilibrium with calcite, the Ca concentration of ~3 mM matches experimental data^64^. If the solution is allowed to equilibrate with rhodochrocite as well as calcite, the resulting Mn/Ca molar ratio is about 0.05 (input file in SI), which is very similar to the calculations at 25 °C and low ionic strength (Fig. 4). However, the uncertainties at reservoir conditions are larger and more experimental input would be valuable. For example, based on its solubility product in the llnl database, Mn(II)(OH)_2_ is predicted to be significantly undersaturated. However, more oxidised Mn species are highly insoluble. Although Mn(II) oxidation rate is slow at ambient temperature, heterogeneous oxidation can be strongly temperature dependent (e.g. ref. 70). Thus, if Mn(II) is injected in aerobic solutions or into reservoirs that are aerobic because of prior injections, Mn(III) or Mn (VI) oxides could form and could block pores.

## Conclusions

Density functional theory calculations predict how solid-fluid interfacial energies change for water and organic phases on calcite surfaces as a result of divalent cation substitution. Calcite becomes significantly more hydrophilic when divalent transition metal ions (Mn, Fe, Co, Ni, Cu and Zn) incorporate into the calcite surface and it becomes more hydrophobic when Sr, Ba and Pb incorporate. We found a qualitative correlation showing that the more strongly hydrated the substituted ion is, the more water wet the surface becomes. The incorporation of transition metals is exothermic and therefore much lower concentrations are required to alter calcite wettability than for Mg. This means that even small concentrations of trace metals can have a large impact on biomineralisation rates and provides one explanation for why biomineral formation sometimes goes wrong, if extraneous ions sorb on or in the mineral surface. This would upset the organism’s desired wetting properties.

The results of these simulations and the consistency with previously published experimental data strongly suggests that marine organisms use Mg and trace amounts of Sr, Ba and other metals in sea water to modify surface free energy during biomineralisation, to turn on and off the effect of organic compounds, such as polysaccharides and other biomolecules. This is an important result.

We tested change in surface behaviour as a result of exposure to model organic phases, such as carboxylate, nitrogen base, alcohol and aromatic hydrocarbon. The carboxylate bonded more strongly than water and confirms previous studies of acidic molecules being able to render calcite more hydrophobic and to be able to alter calcite growth kinetics. An important point is that for organic phases with a mole fraction of up to 1% acids, alcohols or bases, all the transition metals render calcite more hydrophilic.

If H_2_S is present in the system, our simulations predict that sulphide minerals would effectively scavenge the strongly hydrating transition metals (Co, Ni, Cu and Zn). This would make solution concentrations so low that wettability alteration on calcite would be negligible. The presence of CO_2_ gas plays only a minor role.

In terms of enhanced oil recovery, several of the transition metal ions would increase water wetting. For low temperature carbonate oil reservoirs, such as Middle East limestone, we propose divalent Mn as the best candidate because of its low hydration energy and its insensitivity to the presence of H_2_S and the composition of the oil. For higher temperature reservoirs, such as North Sea chalk, an additional candidate is divalent Fe. In the absence of H_2_S, Zn and Ni are also potential candidates. They produce the largest change in wettability but at lower temperatures, slow dehydration rates could hinder sorption of Ni and if H_2_S is present, Ni and Zn sulphide phases would decrease their concentration in the water flood, minimizing activity on pore surfaces.

Our results are general, with potential applications also in other systems. For example, by changing the interfacial energy of particles in contaminated soil or groundwater aquifers, improved remediation strategies might be possible. An important aspect of the results is that the methods we have developed and the mechanisms that have been revealed open new possibilities for atomistic modelling. It has improved nanometre scale understanding about biomineralisation. Our methods for predicting changes in solid-fluid interfacial energies are particularly interesting, considering recent developments for determining liquid interfacial energies from first principles^71^. The possibility to predict liquid-liquid and solid-liquid interfacial energies using first principles suggests that more detailed simulations of biomimetic systems are within reach, with implications for developing new functional materials.

## Additional Information

**How to cite this article**: Andersson, M. P. *et al*. Modelling how incorporation of divalent cations affects calcite wettability–implications for biomineralisation and oil recovery. *Sci. Rep*. **6**, 28854; doi: 10.1038/srep28854 (2016).

## Supplementary Material

Supplementary Information

## Figures and Tables

**Table 1:** Energy of substitution for cation exchange into the 1^st^ and 2^nd^ layer of calcite {10.4}, calculated using Eq. 6.

Ion	Substitution energy top layer of calcite [kJ/mol]	Substitution energy 2^nd^ layer of calcite [kJ/mol]
Mg^2+^	14	22
Sr^2+^	11	12
Ba^2+^	−43	−57
Pb^2+^	−30	−29
Cd^2+^	−43	−44
Mn^2+^	−24	−21
Fe^2+^	−20	−13
Co^2+^	−25	−15
Ni^2+^	−22	−13
Cu^2+^	−44	−27
Zn^2+^	−22	−10

**Table 2:** Adsorption energy, Eads, for water and organic compounds, X, on calcite and substituted calcite from Eq. 2 (organic molecules) and Eq. 3 (water).

Substituted ion (Me)	Water [kJ/mol]	Acetic acid [kJ/mol]	Pyridine [kJ/mol]	Phenol [kJ/mol]	Benzene [kJ/mol]
calcite only	−102	−139	−83	−79	−63
Mg^2+^	−121	−146	−80	−84	−51
Sr^2+^	−85	−138	−84	−79	−65
Ba^2+^	−70	−130	−82	−83	−101
Pb^2+^	−61	−112	−66	−85	−56
Cd^2+^	−99	−130	−85	−61	−61
Mn^2+^	−103	−131	−78	−66	−53
Fe^2+^	−109	−136	−87	−70	−52
Co^2+^	−86	−126	−77	−83	−28
Ni^2+^	−119	−143	−104	−71	−51
Cu^2+^	−103	−125	−87	−71	−47
Zn^2+^	−111	−133	−78	−65	−45

**Table 3:** Difference in adsorption energy, ΔΔE_ads_, between water and organic compounds, X, for substituted calcite and pure calcite, calculated using Eq. 5.

Substituted ion (Me)	Acetic acid [kJ/mol]	Pyridine [kJ/mol]	Phenol [kJ/mol]	Benzene [kJ/mol]
calcite only	0	0	0	0
Mg^2+^	12	22	14	31
Sr^2+^	−16	−18	−17	−19
Ba^2+^	−23	−31	−36	−69
Pb^2+^	−13	−24	−47	−34
Cd^2+^	6	−4	15	1
Mn^2+^	10	6	13	12
Fe^2+^	11	3	16	18
Co^2+^	−2	−9	−19	20
Ni^2+^	13	−4	26	29
Cu^2+^	15	−2	9	17
Zn^2+^	15	14	23	27

Negative values correspond to a more hydrophobic surface, whereas positive values correspond to a more hydrophilic surface.

**Table 4:** Predicted oil-water interface composition for some model organic compounds, where the number of polar functional groups varies.

Oil component	Typical oil phenol = 0.1% acetic acid = 0.1% pyridine = 0.1%	High acid oil phenol = 1% acetic acid = 1% pyridine = 0.1%	High base oil phenol = 0.1% acetic acid = 0.1% pyridine = 1%
Hexane	29.9%	11.6%	32.9%
Benzene	41.4%	16.0%	45.4%
Acetic acid	25.6%	64.7%	9.9%
Phenol	1.0%	7.6%	1.0%
Pyridine	0.8%	0.1%	10.8%

The rest is nonpolar oil, half benzene and half hexane. The values for the energy of transfer of the molecule from bulk organic phase to the oil-water interface, that were used to calculate the interface composition are presented in Table SI-3.

**Table 5:** The extent of divalent cation substitution into the first layer of calcite and the resulting contact angle change (using Eqs 7 and 8), assuming an initial contact angle of 120°.

Ion	% substituted ions first layer CO_2_ [10^−3.5^ to 1 bar]	Δθ (°) CO_2_ [10^−3.5^ bar]	% substituted ions first layer CO_2_ [10^−2^ bar] and H_2_S [10^−3.5^ bar]	Δθ (°) CO_2_ [10^−2^ bar] and H_2_S [10^−3.5^ bar]
Mg^2+^	2	−6	2	−6
Mn^2+^	25	−38	25	−38
Fe^2+^	25	−47	12	−24
Co^2+^	25	−10	0	0
Ni^2+^	25	−68	0	0
Cu^2+^	25	−55	0	0
Zn^2+^	25	−70	0	0

The pH in the simulations was 7.5 (Fig. 4b). More than 25% sorption might occur for Mn, Fe, Co, Ni and Zn but our DFT set up capped substitution at 1 of the 4 available cation surface sites.
