# Supplementary material for: Modelling how incorporation of divalent cations affects calcite wettability–implications for biomineralisation and oil recovery
Source: Sci Rep. 2016 Jun 29;6:28854. doi: 10.1038/srep28854 (PMC4926276; doi:10.1038/srep28854)
Supplement: Supplementary Information [file srep28854-s1.pdf]

Supporting information for:

Modelling how incorporation of divalent cations affects calcite wettability – implications for biomineralisation and oil recovery.

M.P. Andersson[1*], K. Dideriksen[1], H. Sakuma[1,2], S.L.S. Stipp[1]

[1] *Nano-Science Center, Department of Chemistry, University of Copenhagen, Denmark,*
[2] *current address: Environmental Remediation Unit, National Institute for Materials Science, Japan*

* corresponding author: ma@nano.ku.dk

Additional computational details

COSMO-RS calculations
The pK_a for water and acetic acid adsorbed on substituted calcite slabs was calculated using COSMO-RS[1] and the COSMOtherm[2,3] program. We used Turbomole v6.5 [4,5], the BP [6] [7] functional and the TZVP basis set [8], and an infinite dielectric constant for the COSMO implicit solvent. The BP_TZVP_C30_1301 parameterization was used in all COSMO-RS calculations. For the surface pK_a simulations, we used 80 atom clusters to represent the calcite surface and relaxed only the 10 atoms of the surface that are not near an edge. This procedure gave a converged pK_a for water on unsubstituted calcite [9]**.** An example of the system is shown in Figure SI-1.

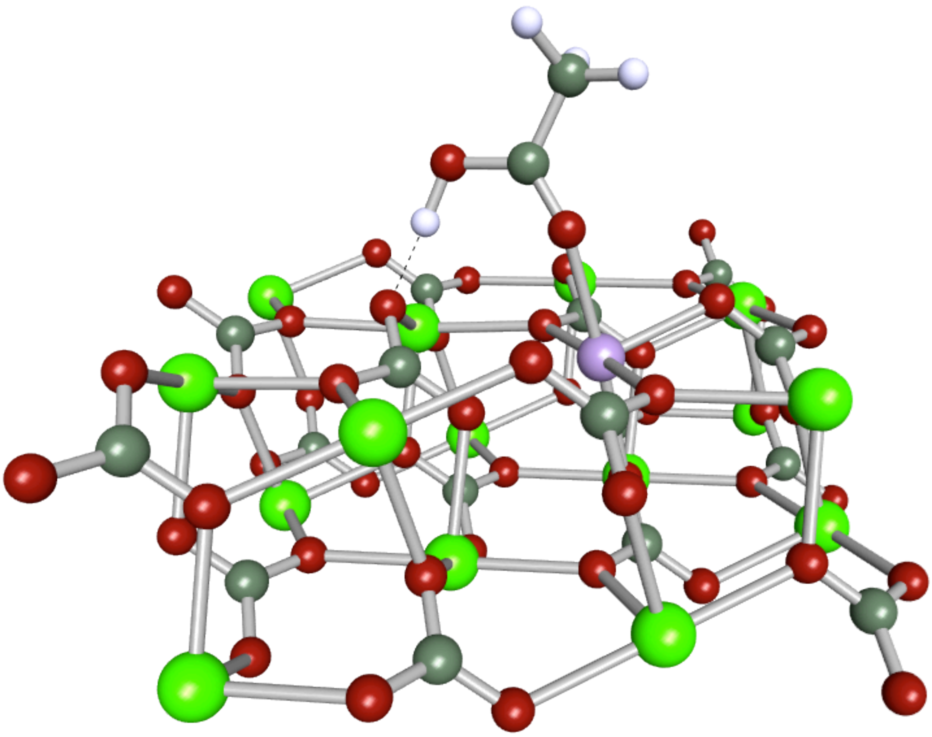

Figure SI-1. Cluster model (80 atoms) used for adsorption of acetic acid on Mn substituted calcite. Ca is represented with bright green spheres; C with grey; O, red; H, white and Mn, purple.

Additional results

The change in adsorption energy , ΔE_{ads}, resulting from ion substitution, is shown in Table SI-1. The most obvious trend is that a surface where Mg or any transition metal substitutes, adsorption of water is strongly favoured over benzene, which makes sense, considering the free energy of hydration.

Table SI-1: The change in adsorption energy, ΔE_{ads}, between water and organic compounds on a calcite surface where divalent cations are substituted, compared with pure calcite. Energies are in kJ/mol.

**Substituted ion (I)**	**Water (monolayer, 4 molecules)**	**Acetic acid**	**Pyridine**	**Phenol**	**Benzene**
**Mg^{2+}**	-19	-7	3	-5	12
**Sr^{2+}**	17	1	0	0	-2
**Ba^{2+}**	32	9	1	-4	-37
**Pb^{2+}**	40	27	17	-7	7

**Cd^{2+}**	2	9	-1	18	3
**Mn^{2+}**	-1	9	5	13	11
**Fe^{2+}**	-7	4	-4	9	11
**Co^{2+}**	15	14	6	-4	35
**Ni^{2+}**	-17	-4	-21	8	12
**Cu^{2+}**	-1	14	-3	8	16
**Zn^{2+}**	-9	6	5	14	18

The free energies for transferring molecules from oil to the oil-water interface are shown in Table SI-2. The trends are very similar to our previous study [10] but the values are slightly different. Some reasons are that we are using different molecules and different oil composition for this work and also that the COSMO-RS parameterization is new and slightly improved. The values in Table SI-2 were used to generate equilibrium constants to describe the composition at the oil-water interface for the simulation in the main paper.

Table SI-2: Free energy for transfer, ΔE_{trans}, from the bulk oil to the oil-water interface for some model oil compositions where the balance is 50% hexane and 50% benzene. Energies are in kJ/mol.

	**Typical oil**<br>**phenol = 0.1%**<br>**acetic acid = 0.1%**<br>**pyridine = 0.1%**	**High acid oil**<br>**phenol = 1%**<br>**acetic acid = 1%**<br>**pyridine = 0.1%**	**High base oil**<br>**phenol = 0.1%**<br>**acetic acid = 0.1%**<br>**pyridine = 1%**
**Hexane**	4.6	4.6	4.6
**Benzene**	3.8	3.8	3.8
**Acetic acid**	-10.4	-9.3	-7.8
**Phenol**	-4.5	-4.0	-2.1
**Pyridine**	-1.7	0.6	-2.3

The pKa values for water and acetic acid on calcite {10.4} are presented in Table SI-3. pKa for both water and acetic acid adsorbed on calcite are lower than their values in bulk water, and there is little variation for the acetic acid.

Table SI-3. pKa values predicted for water and acetic acid, adsorbed on calcite {10.4}.

**Ion**	**pKa of adsorbed water**	**pKa of adsorbed acetic acid**
**Ca^{2+}**	12.8	3.0
**Mg^{2+}**	12.2	3.6
**Sr^{2+}**	11.4	2.7
**Ba^{2+}**	12.1	1.6
**Pb^{2+}**	11.8	2.2
**Cd^{2+}**	11.8	3.2
**Mn^{2+}**	11.1	3.4
**Fe^{2+}**	9.5	3.8
**Co^{2+}**	9.4	3.0
**Ni^{2+}**	11.3	3.2
**Cu^{2+}**	9.9	2.2

**Zn^{2+}**	11.0	3.4

References

1 Klamt, A., Jonas, V., Burger, T. & Lohrenz, J. C. W. Refinement and parametrization of COSMO-RS. *J. Phys. Chem. A* **102**, 5074-5085 (1998).

2 Eckert, F. & Klamt, A. Fast solvent screening via quantum chemistry: COSMO-RS approach. *AIChE J.* **48**, 369-385 (2002).

3 COSMOtherm Version C3.0, Release 13.01 v. C3.0, Release 13.01 (COSMOlogic GmbH & Co. KG, Leverkusen, Germany, 2013).

4 TURBOMOLE V6.3 2011, a development of University of Karlsruhe and Forschungszentrum Karlsruhe GmbH, 1989-2007, TURBOMOLE GmbH, since 2007.

5 Ahlrichs, R., Bar, M., Haser, M., Horn, H. & Kolmel, C. Electronic-structure calculations on workstation computers - the program system TURBOMOLE. *Chem. Phys. Lett.* **162**, 165-169, doi:10.1016/0009-2614(89)85118-8 (1989).

6 Becke, A. D. Density-functional exchange-energy approximation with correct asymptotic behavior. *Phys. Rev. A* **38**, 3098-3100 (1988).

7 Perdew, J. P. Density-functional approximation for the correlation energy of the inhomogeneous electron gas. *Phys. Rev. B: Condens. Matter* **33**, 8822-8824 (1986).

8 Schafer, A., Horn, H. & Ahlrichs, R. Fully optimized contracted gaussian-basis sets for atoms Li to Kr. *J. Chem. Phys.* **97**, 2571-2577 (1992).

9 Andersson, M. P. & Stipp, S. L. S. How acidic is water on calcite? *The Journal of Physical Chemistry C* **116**, 18779-18787, doi:10.1021/jp304671k (2012).

10 Andersson, M. P., Olsson, M. H. M. & Stipp, S. L. S. Predicting pKa and stability of organic acids and bases at an oil-water interface *Langmuir* **30**, 6437-6445, doi:10.1021/la5008318 (2014).
